# Effect of Flavored on! Nicotine Pouch Products on Smoking Behaviors: Protocol for a Sequential, Multiple Assignment, Randomized Controlled Trial

**DOI:** 10.2196/56565

**Published:** 2024-06-21

**Authors:** Hui G Cheng, Jed E Rose, Joshua L Karelitz, David R Botts, Tanaia L Botts, Perry N Willette, Gal Cohen

**Affiliations:** 1 Altria Client Services, LLC Richmond, VA United States; 2 Rose Research Center, LLC Raleigh, NC United States

**Keywords:** nicotine pouches, flavored products, smoking reduction, tobacco harm reduction, randomized controlled trial

## Abstract

**Background:**

Cigarette smoking is a leading cause of morbidity and mortality. For adults who smoke cigarettes and cannot or will not quit smoking, smoke-free products, such as nicotine pouches, have been recognized as a potential alternative to smoking combusted cigarettes to reduce harm due to cigarette smoking. The role of flavors in these smoke-free products in tobacco harm reduction has not been fully understood.

**Objective:**

This study evaluates the effect of flavors in *on!* nicotine pouch products (research products) in the reduction of cigarette smoking among adults who smoke cigarettes in their natural environment.

**Methods:**

This study uses a sequential, multiple assignment, randomized trial design. Approximately 400 eligible adults who smoke cigarettes will be enrolled and randomized to have access to either the Original (unflavored) *on!* nicotine pouch product only or a complete flavor profile (ie, Berry, Cinnamon, Citrus, Coffee, Mint, Original, and Wintergreen) of *on!* nicotine pouch products. After 3 weeks, participants in the Original-only arm will be randomized again, with half remaining in the Original-only arm and half having access to the complete flavor profile for another 3 weeks. Primary outcomes are expired-air carbon monoxide (CO) levels. Secondary outcomes are self-reported cigarette consumption and CO-verified cigarette abstinence.

**Results:**

Recruitment and data collection started in September 2023 and is projected to last until March 2025. We anticipate completing the data analysis in 2025. As of May 2024, we have enrolled 314 participants.

**Conclusions:**

This study will provide empirical evidence about the effect that flavor availability in smoke-free products may have in reducing cigarette smoking.

**Trial Registration:**

ClinicalTrials.gov NCT06072547; https://clinicaltrials.gov/study/NCT06072547

**International Registered Report Identifier (IRRID):**

DERR1-10.2196/56565

## Introduction

Smoking tobacco cigarettes is the leading cause of preventable morbidity and mortality in the United States, with approximately half a million attributable deaths each year [1,2]. The vast majority of smoking-related diseases are caused by inhaling harmful and potentially harmful constituents (HPHCs) present in tobacco smoke as a result of combustion [3,4]. While not entirely risk free, nicotine is not directly responsible for most of the harm caused by smoke exposure from combusted tobacco products [5]. The US Food and Drug Administration (FDA) and public health experts acknowledge that nicotine and tobacco products exist along a continuum of risk, ranging from combusted products posing the highest risk and noncombusted products at the lower end [5-8]. Complete tobacco cessation is the best option for reducing smoking-related morbidity and mortality for the 28.3 million adults who smoke cigarettes in the United States [9]. Nonetheless, tobacco cessation is often a protracted process that can span over multiple years for many adults who smoke cigarettes [10]. For adults who smoke cigarettes who cannot or are unwilling to stop smoking cigarettes, switching completely or replacing most of their combusted cigarettes with smoke-free nicotine products may be a viable harm-reduction option [11].

Oral tobacco-derived nicotine products are a relatively new category among the growing field of smoke-free nicotine products. Within this growing category are nicotine pouches (NPs), which contain pharmaceutical-grade nicotine derived from tobacco (but are tobacco-leaf free) along with flavors and other food-grade ingredients [12]. These products are commercially available in the United States in a wide variety of flavors (eg, mint, fruit, tobacco, coffee, and unflavored), with nicotine levels typically ranging 1.5-8.0 mg (with some brands offering levels up to 50 mg) [12-14]. NPs typically contain significantly lower levels of HPHCs than cigarette smoke [15,16], and switching from combusted cigarettes to these products has been shown to significantly reduce HPHC exposure to the user [17], positioning these products to serve as a potential reduced-harm option for adults who smoke cigarettes. Among the few studies assessing the longitudinal use of NPs among adults who smoke cigarettes, initial evidence suggests that adults who smoke cigarettes may switch to these smoke-free products or use them to help reduce cigarette consumption.

To date, we are aware of only 2 published studies of switching behavior or smoking reduction over time among adults who smoke cigarettes provided with NPs [13,18]. Campbell et al [18] provided participants with free choice among NPs in 2 flavors—mint or citrus—in a single nicotine level (4 mg) to use ad libitum for 6 weeks. At the end of the study, the majority of participants (79/97, 82%) reduced cigarette consumption, with 16% (15/97) reducing by ≥50% and 3.1% (3/97) self-reporting stopping smoking. No switching behavior outcomes were reported. This pilot study was intended to refine procedures and was not adequately powered to detect significant effects of using the research product on smoking reduction outcomes. Becker et al [13] examined longitudinal ad libitum use of NPs over 6 weeks among adults who smoke cigarettes (n=399). When provided with NPs in 7 flavors (ie, cinnamon, citrus, coffee, berry, mint, original, and Wintergreen) and 5 nicotine levels (ie, 1.5, 3, 3.5, 4, and 8 mg), 27% of adults who smoke cigarettes completely switched from cigarettes to NPs. Additionally, 39% reduced cigarette consumption by ≥50%, and 24% reduced their consumption by up to 49%. Interestingly, Becker et al [13] reported a positive association between the number of flavors used and the magnitude of cigarette reduction. Overall, these studies provide strong initial evidence that, in the context of free product provision, adults who smoke cigarettes are willing to switch to NPs, and those who do not completely switch can have meaningful reductions in their cigarette consumption when using NPs. Although they provided some initial insights about the potential role of flavors, the observational nature of these studies (eg, uncontrolled, no comparator groups, and no randomization) preclude the inference of causal relationships between NP use (regardless of flavor) and cigarette reduction.

The examination of available NP sales data shows that flavored varieties (eg, mint and fruit) consistently outsell nonflavored or “Original” varieties [19]. However, these data do not provide information on consumers’ concomitant use of other tobacco products (ie, switching or cigarette reduction). We designed this protocol to fill the knowledge gap and provide important information on the role of NP flavors on switching behavior and cigarette reduction among adults who smoke cigarettes (ClinicalTrials.gov NCT06072547). Specifically, this study uses a sequential, multiple assignment, randomized trial (SMART) design [20,21] to assess the effect of the availability of flavored (vs unflavored) *on!* brand NPs—holding nicotine level constant at 4 mg per pouch—on the smoking behavior of adults who smoke cigarettes over 6 weeks. Our purpose in this paper is to describe the protocol for a SMART study involving sequential randomization of adults who smoke cigarettes to provide evidence from an experimental study to advance the understanding of the role of NP flavors in smoking reduction. Our study objectives are to assess the following:

1. The overall effect of the availability of a complete flavor profile of the research products on smoking reduction among adults who smoke cigarettes.
2. The effect of the availability of the complete flavor profile of the research products on smoking reduction maintenance among participants who reduce their cigarette consumption ≥50% at week 3 (ie, responders).
3. The effect of the availability of the complete flavor profile of the research products on the smoking reduction at week 6 among participants who did not reduce their cigarette consumption ≥50% at week 3 (ie, nonresponders).
4. The effect of early versus delayed availability of complete flavor profile of the research products on smoking reduction.

We hypothesize that access to a complete profile of flavored NP products will result in greater reduction, including greater persistence of reduction, in exposure to a smoking-related toxicant (ie, carbon monoxide [CO]) and cigarette consumption. With objectives 1-3, we intend to assess the hypothesis from 3 different angles (ie, overall effect, maintenance of reduction among responders, and additional reduction among nonresponders). With objective 4, we seek to explore the potential effect of a delayed access to flavored products, which may have regulatory implications for the speed of decision-making on flavored products. We consider objective 4 to be exploratory because it is difficult to simulate such a scenario in a trial context with limited follow-up time.

## Methods

### Study Design

#### Overview

**Figure 1:**
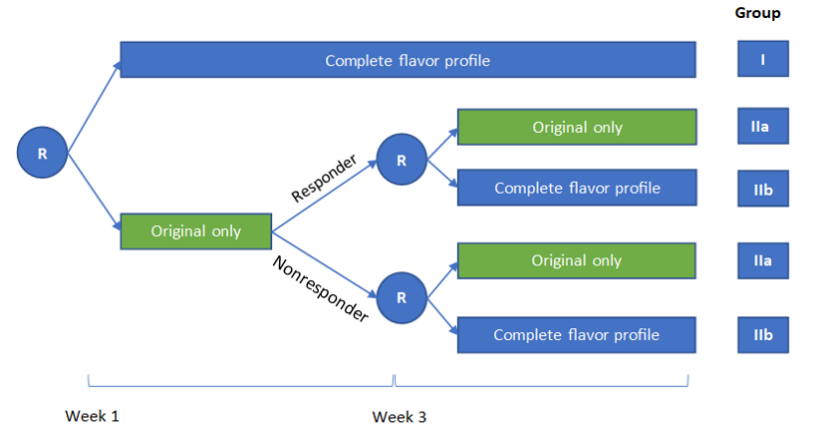
Depiction of study design. Responders are participants who have a ≥50% reduction in cigarette consumption at week 3 (relative to baseline assessment); nonresponders are participants who have a <50% reduction in cigarette consumption at week 3 (relative to baseline assessment). R: random assignment.

This study will use an open-label randomized controlled trial design to evaluate the impact of availability of flavored versus nonflavored (ie, Original) *on!* 4-mg NP products on cigarette smoking among adults who smoke cigarettes. This study involves a remote screening visit, a baseline assessment period (3-7 days), 6 weeks of at-home product use, and a 6-month posttrial follow-up survey. Figure 1 provides a depiction of the study design (baseline assessment prior to week 1 and 6-month follow-up are not shown).

#### Randomization

Approximately 400 participants will be recruited over an approximately 7-month period. After confirming eligibility and obtaining informed consent, participants will be randomized into 3 groups varying only by the availability of research product flavors. Group I (n=150) will have access to the complete flavor profile of the research products (ie, Berry, Citrus, Cinnamon, Wintergreen, Mint, Coffee, and Original). Group IIa (n=125) will have access to only the Original variety for the entire 6-week trial period. Group IIb (n=125) will have access to only the Original variety for the first 3-week period and will be provided access to the complete flavor profile for the second 3-week period. We chose to randomize participants to these 3 groups at study onset rather than have sequential randomizations (ie, first randomize to the full flavor profile or Original-only arm, then rerandomize those in the Original-only arm to either the full flavor profile or remain with the Original-only arm) to simplify the logistics of randomization.

#### Baseline Assessment

After randomization and prior to week 1, participants will be asked to track their daily cigarette consumption via a web-based daily diary and provide ≥2 biometrically verified expired-air CO readings via Bluetooth-enabled remote CO monitor (iCOquit Smokerlyzer, Bedfont Scientific) over a period of 3-7 days. These initial data will serve as baseline cigarette consumption and CO for subsequent comparisons.

#### Weeks 1-6

In week 1, participants randomized into Group I will enter a 7-day period, during which they will receive 7 packs (20 pouches per pack) of research product (1 of each flavor) and instructed to try each flavor. This trial period is intended to allow participants to identify their preferred flavor(s) to use over the remainder of the study. After the trial period in week 1, Group I participants will complete 5 additional weeks of ad libitum product trial of research products (ie, weeks 2-6) with free choice of all flavors (up to 7 packs per week in any flavor combination of their choosing).

Likewise, following the baseline assessment, participants randomized into Group IIa and Group IIb will be provided with the Original variety of research products to use ad libitum for weeks 1-3 (up to 7 packs per week). At the end of week 3, participants in Group IIa will continue with the Original variety of research product for the remaining 3 weeks of the study (ie, weeks 4-6), and those in Group IIb will be provided access to all flavors of the research product. In week 4, Group IIb will have a 7-day trial period, during which they will receive 7 packs of research product (1 of each flavor) and instructed to try each flavor. As with Group I, this trial period is intended to allow Group IIb participants to identify their preferred flavor(s) to use over the remainder of the study. After the week-4 trial period, Group IIb will complete 2 weeks of ad libitum product trial of research products with free choice among all flavors (up to 7 packs of any combination per week).

Participants will be invited to complete a 6-month follow-up survey to gain insights about their tobacco use and related factors after the end of the product trial. This study will involve approximately 7 weeks (including the baseline period) of study participation in a home use test with daily surveys, weekly surveys, and a 6-month follow-up survey after the trial period.

### Inclusion and Exclusion Criteria

The study sample will include approximately 400 adults who smoke cigarettes, between 22 and 65 years of age who have smoked an average of at least 5 commercial brand cigarettes per day for 12 months prior to signing consent. Specific inclusion and exclusion criteria are listed in Textbox 1.

**Inclusion and exclusion criteria.**

**Inclusion criteria**

- Has signed the informed consent form (ICF) and is able to read and understand the information provided in the ICF.
- Healthy adults who smoke cigarettes and are 22-65 years of age at screening.
- Smokes an average of at least 5 cigarettes per day for the last 12 months.
- Does not intend to use a Food and Drug Administration–approved treatment for nicotine dependence within the next 60 days (as assessed at screening).
- Responds “Yes” to “Are you interested in replacing combustible cigarettes with a smoke-free tobacco product?” when asked at screening.
- Willing and able to comply with the requirements of the study.
- Owns a smartphone with SMS text messaging and data capabilities compatible with necessary surveys.

**Exclusion criteria**

- Participant enrollment numbers met (in subgroup or entire study).
- Participant, or their first-degree relative (eg, parent, sibling, child, or spouse) or household member, is a current or former employee of the tobacco or e-vapor industry.
- Participant, or their first-degree relative (eg, parent, sibling, child, or spouse) or household member, is a named party or class representative in litigation involving a tobacco or e-vapor company.
- Participant, or their first-degree relative (eg, parent, sibling, child, or spouse) or household member, is a current or former employee of a marketing consultant, market research firm, advertising or promotions agency, TV or radio station, magazine or newspaper, government regulatory agency or public policy advocacy group, or law firm or legal department of a company.
- Participant self-reports being “in poor health.”
- Participants of childbearing potential (CBP) who have a positive pregnancy test (as assessed at screening) or are nursing or planning to become pregnant during their participation.
- Participant has an allergy or sensitivity to menthol or menthol-containing products or phenylalanine.
- Participant has any other self-reported health restrictions.
- Participant self-reports cardiovascular disease, cancer, or diabetes or is being treated for high blood pressure.
- Participant self-reports periodontal disease, gum disease or bleeding, open mouth sores, or ulcers.
- Participant self-reports as wanting to stop using tobacco products in the next 60 days.
- Participant has participated in 1 tobacco research study in the past 30 days or a tobacco research study lasting 2 weeks or longer in the past 90 days.
- Participant is unable to read, speak, or understand English.
- Participants who ever used at least a pack of nicotine pouch products or currently uses nicotine pouch products.
- Participant who smokes marijuana more than once a week.
- Heterosexually active participants of CBP (not sterilized by tubal ligation, oophorectomy, hysterectomy, or other surgical methods, or postmenopausal) who do not agree to practice medically appropriate methods of birth control (or remain abstinent) during the course of the trial and for 30 days after the last use of research product. Medically acceptable methods of birth control include vasectomy, vaginal diaphragm with spermicide, intrauterine device, hormonal birth control (oral, injected, patch, or implanted), condom with spermicide, or sponge with spermicide.
- Taking psychoactive medications (eg, antipsychotics or mood stabilizers).
- Cannot participate in the study for any reason (eg, medical, psychiatric, or social reason) as judged by the investigator or designated medical staff based on all available information from the screening period.

### Ethical Considerations

The study protocol and informed consent form (ICF) has been approved by Advarra Institutional Review Board (IRB; Pro00072765). Study conduct will follow the principles set forth by the Belmont Report and, where applicable, guidelines established under 21 Code of Federal Regulations (CFR) § 50 and 56. In addition, study conduct will be in accordance with the ethical principles that have their origin in the Declaration of Helsinki and that are consistent with Good Clinical Practice (GCP) and applicable regulatory requirements. The study must be conducted in accordance with the regulations of the US FDA as described in 21 CFR 50 and 56, applicable laws, and the IRB requirements.

Informed consent will be obtained from all participants remotely through an electronic consent process. Participants will be required to verify that they understand each section of the ICF by checking a box. They will provide their consent at the end of the document by checking a box after reading the consent statement. Electronic copies of the ICF are made available for the participants to download and reference at any time. Participants will be informed that their participation is completely voluntary, and they may discontinue their participation at any time for any reason.

After providing consent, participants will be asked to upload a copy of their government-issued ID for verification. We will use a multistep process for age verification, validation of the uploaded government-issued identification, and to ensure that the person matches the photo on the ID. This process will be conducted by software and research staff. Software will initially determine whether the uploaded ID is valid, verify the individual’s age, and compare previously submitted information against what is detected on the uploaded ID. Any discrepancies at this point will be reviewed by research staff. If the uploaded ID is expired, blurred, or otherwise not valid, the potential participant will be given the opportunity to resubmit and continue the screening process. For those who pass the initial software-level review, research staff will verify that the face on the ID matches the individual who joins the telemedicine session. We rely on human review for this final step for the safety of minors and due to limitations in currently available software.

### Recruitment

Participants will be recruited through IRB-approved, study-specific recruitment advertisements or from the Rose Research Center volunteer database, an Advarra IRB-approved generic volunteer database. Participants will be contacted with IRB-approved materials. These documents will include a brief description of the study and information on how to prescreen for participation. This study will recruit remotely across the United States in North Carolina, Ohio, Georgia, Florida, Michigan, South Carolina, Tennessee, and Pennsylvania. Participant recruitment will be guided by the distribution of demographic characteristics shown in Table 1.

**Table 1:** Demographic distribution of adults who smoke cigarettes based on National Survey on Drug Use and Health (NSDUH) 2021 data.

Demographic subgroup					Prevalence in US adult smoker population, %^a^			Corresponding average expected cell size given (n=400), n
**Sex and age (years)**								
	**Male**			53.9			216	
		21-34	11.0			44		
		35-44	13.2			53		
		45-49	6.1			24		
		50-64	23.6			94		
	**Female**			46.1			184	
		21-34	9.6			38		
		35-44	12.1			48		
		45-49	5.8			23		
		50-64	18.7			75		
**Race or ethnicity**								
	White non-Hispanic			77.7			311	
	Black non-Hispanic			8.1			32	
	Other non-Hispanic			6.3			25	
	Hispanic			7.9			32	
**Education**								
	Less than College			60.4			242	
	Some college			30.20			121	
	College+			9.40			38	
**Menthol preference**								
	Menthol			36.20			145	
	Nonmenthol			63.80			255	

^a^Estimates were based on weighted proportions.

### Study Procedure

This study involves a multistep screening process, a baseline assessment period (3-7 days), 6 weeks of at-home product use, and a 6-month posttrial follow-up survey. During recruitment, participants will sign the ICF, upload a picture of their government-issued ID, complete web-based screening surveys, and download eResearch Advance Science from Home to their smartphones.

All who pass the initial web-based screening will have a CO monitor sent to their home address. At this point, male participants are able to schedule their baseline telehealth session and continue the screening process. Female participants are sent a urine pregnancy test with instructions for completing and uploading results, which are reviewed and confirmed by study staff. Those with confirmed negative pregnancy test results are then able to schedule the baseline telehealth session.

At the baseline telehealth session, participants will be further assessed for eligibility with additional inclusion or exclusion criteria, including a biometrically verified expired-air CO reading. If eligible, they will be randomized to Group I or Group IIa/IIb. During the 3- to 7-day baseline assessment period, participants will provide CO readings every other day and record their cigarette consumption in web-based daily diary survey. Upon the completion of the baseline assessment period, participants will begin week 1 when they receive the research product to use in their natural environment, following group configurations shown in Figure 1. During weeks 1-6, participants will respond to daily diary surveys to report their use of the research product (by flavor, if applicable), cigarette consumption, and use of other tobacco products. They will request and receive research products weekly. They will also be alerted to provide pseudorandom biometrically verified CO readings once every 3 days. At the end of week 3, female participants of childbearing potential will be asked to provide urine pregnancy test results; those with positive results (ie, pregnant) will be discontinued from the study and referred to their primary care provider. Six months after the study ends, participants will be recontacted to complete a follow-up survey with questions related to tobacco-use behaviors (including cigarette smoking and *on!* NP use), reasons to use or not use *on!* NPs, risk perceptions of use of a variety of tobacco products at the category level (eg, cigarettes, smokeless tobacco, and NPs), and respiratory symptoms. The main purpose of the 6-month follow-up survey is to collect information on participants’ posttrial tobacco use behaviors when they are no longer provided with *on!* NPs. None of our research questions rely on the 6-month follow-up survey.

Participants will be presented with the following debriefing statement at the conclusion of the study (ie, at the end of Week 6) or upon discontinuation of participation (ie, withdrawal from the study):

We would like to emphasize that in conducting this research, we were not trying to market, sell or promote a tobacco or nicotine product to you. Finally, oral tobacco-derived nicotine products should never be viewed as an alternative to quitting all tobacco products.

Research will be conducted in the context of participants’ natural environment (ie, home use test). Research products will be shipped to participants weekly.

Participants will receive compensation after successful completion of each study milestone, including the completion of each daily survey, weekly survey, and CO measurement collection throughout the study. An end-of-study bonus will be awarded if the participant completes the entire study.

### Outcome Measures

Primary outcomes of this study are (1) at least 50% reduction in expired-air CO readings from baseline (yes or no) and (2) expired-air CO as a continuous variable (averaged across the week of interest).

Secondary outcomes include (1) at least 50% reduction in self-reported cigarette consumption from baseline (yes or no), (2) CO-verified smoking abstinence (ie, self-reporting no cigarettes smoked in past 7 days and CO<6ppm [22]), and (3) self-reported number of cigarettes smoked (averaged across the week of interest).

Participants who meet the ≥50% cigarette reduction (based on self-reported cigarette consumption) threshold will be identified as “Responders”; those unable to meet this threshold will be identified as “non-Responders.”

We chose to use expired-air CO values in primary outcomes because it is an objective measure not susceptible to recall bias, demand characteristics, or prevarication. Further, expired-air CO closely reflects actual smoke exposure and would not lead to an erroneous conclusion of reduced exposure that results from more intensive smoking of fewer cigarettes as compared to self-reported cigarette consumption. Nonetheless, expired-air CO measures are not without limitation. Factors other than cigarette smoking, such as exposure to environmental pollutants, second-hand smoke, vehicle exhaust, etc, can contribute to elevated expired-air CO, and vigorous exercise can lead to lower expired-air CO. In this study, we include both expired-air CO (taken twice a week) and self-reported cigarette consumption (daily) to provide robust assessments of smoking behavior.

### Analytic Plan

A description of recruitment will be reported, including the number of participants who are recruited, pass screening, are randomized, complete weeks 1-3, enter week 4, complete the trial (ie, weeks 1-6), and complete the 6-month follow-up.

We will use descriptive statistics to characterize the distribution of demographic variables, tobacco use history (ie, cigarettes per day, use of other tobacco products, and menthol cigarette preference), and CO at baseline. We will examine the aforementioned demographic variables and tobacco use history variables among responders and nonresponders for potential imbalance. If such imbalance is discovered, respective variables will be adjusted in the analysis as covariates.

The analysis will be conducted on an intention-to-treat basis. Primary outcome variables include a binary (yes or no) variable indicating at least 50% reduction in CO readings from baseline and a numeric variable for expired-air CO. For individuals who drop out of the study prematurely, they will be assumed not achieving at least 50% reduction in CO readings, and their numeric CO reading variable after those last reported will be replaced with their baseline CO readings.

To compare incidence proportions of at least 50% reduction in CO readings between study groups at each time point (week 3 or week 6, depending on the objective), generalized linear regression models with a log link will be used to generate incidence ratios and their 95% CIs. To compare numeric CO readings between study groups at each time point (week 3 or week 6, depending on the objective), linear regression will be used to compare group-level differences in CO readings and their 95% CIs when adjusting for baseline CO reading. We chose to use linear regression adjusting for baseline CO reading over ANOVA for change in CO readings from baseline because the adjustment approach properly corrects for “regression to the mean,” provides greater statistical power, and produces unbiased estimates [23-25].

Similarly, we will use generalized linear regression with a log link for binary secondary outcomes (ie, at least 50% reduction in cigarette consumption based on self-report and CO-verified smoking abstinence). Generalized estimating equations will be used to analyze cigarette consumption based on the daily diary, with cigarette consumption at baseline as a covariate. Specific comparisons are as follows: (1) for objective 1, comparison will be made between (a) Group I and combined Groups II at Week 3 and (b) Group I and Group IIa at Week 6; (2) for objective 2, comparison will be made between Group IIa and Group IIb at week 6 among nonresponders at week 3; (3) for objective 3, comparison will be made between Group IIa and Group IIb at Week 6 among responders at week 3; and (4) for objective 4, comparison will be made between Group I and Group IIb at week 6.

#### Power

Sample size calculation was based on the primary outcomes of the study: at least 50% reduction in expired-air CO readings from the baseline numeric CO readings. Because the Original variety is included in the complete flavors, we do not expect the occurrence of the main outcome to be lower in the complete flavor arms compared to the Original arms, so we will use 1-sided tests with an α level of 0.05 and power of 0.80. Sample size calculation was conducted using Stata 16 (StataCorp).

For objective 1, we assumed an incidence of at least 50% reduction in CO readings of 60% in Group I and 45% in the Original arm at week 3, and incidence of the main outcome among 80% in Group I and 60% in Group IIa at week 6. Based on these values, it was estimated that 136 participants and 64 participants per arm will be needed, respectively. Numeric CO readings will be analyzed as a continuous variable, which typically has higher levels of statistical power [26]. Assuming a mean CO of 6 ppm in Group I and SD of 5 [27], a sample size of 136 per arm will be able to detect a difference of 1.7 between study groups.

For objective 2, we assumed an incidence of at least 50% reduction in CO readings for 70% of those in Group IIb and 45% among the Group IIa at week 6 among the nonresponders in Group II (ie, those who did not reduce cigarette consumption by ≥50% at week 3). Based on these values, we estimated that 48 participants per group will be needed. Assuming 55% of participants in Group II will be nonresponders at week 3; we estimated that a total of 175 participants will be needed in Group II at baseline.

For objective 3, we assumed that 85% in Group IIb and 60% in Group IIa will maintain their responder status at week 6 among responders in Group II (ie, those who reduce cigarette consumption by ≥50% at week 3). Based on these values, it was estimated that 39 participants per group will be needed. Assuming 45% of participants in Group II will be responders at week 3, a total of 174 participants will be needed in Group II at baseline.

Based on these calculations, we will randomly assign 150 participants to Group I and 250 participants to Group II at baseline.

Objective 4 is considered exploratory in nature. Given the above sample size, 125 participants per group will be able to detect a difference between 80% and 67% of at least 50% of CO reduction comparing immediate versus delayed access to complete flavor profile.

## Results

This study was approved by Advarra IRB (Pro00072765) in July 2023. Recruitment and data collection began in September 2023. Recruitment for the trial is projected to end in July 2024 and the entire study in March 2025 (with the 6-month follow-ups). We anticipate publishing study results in 2025. As of May 2024, we have enrolled 314 participants.

## Discussion

### Expected Findings

This study seeks to provide empirical evidence about the causal relationships between availability of flavored smoke-free NP products and the reduction or complete cessation of combusted cigarette smoking, a critical knowledge gap in the literature. In addition to the use of an experimental design, this study was designed to include several features intended to enhance internal and external validity. First, using a SMART design can provide multiple insights about the potential role of flavors in smoking reduction that would not be possible with traditional experimental designs (eg, randomized controlled trials). In other words, using a single study design, we will be able to assess the role of flavors in smoking reduction, maintenance of smoking reduction (among responders), potential added benefit of flavors among nonresponders (ie, the rescue), and the effect of timing (ie, immediate versus delayed access to flavors).

Second, we will remotely collect expired-air CO values. This noninvasive measure provides a validated, objective assessment of recent smoke exposure, not subject to potential bias when relying solely on self-reported cigarette consumption. This is especially relevant when blinding is not feasible as in this study [28,29].

Third, we include the unflavored or Original *on!* NP variety in the complete profile arm to assess the potentially additional effect of flavored products beyond the unflavored or Original variety. This will provide direct evidence from a counterfactual scenario whether there will be any loss of benefit from smoking reduction if a flavor ban was to be implemented for new smoke-free tobacco products, in which case only unflavored or Original products would be allowed in the market. We realize that another scenario relevant to the flavor ban is the removal of all nontobacco flavored smoke-free tobacco products from the market. In this scenario, adults who smoke cigarettes who have replaced their cigarettes with nontobacco flavored smoke-free tobacco products may relapse to smoking or choose to use tobacco-flavored or nonflavored smoke-free products instead. Our study will not provide evidence for this scenario due to considerations of resources and logistics. Future studies with a “constricting” arm where participants have access to the complete profile of smoke-free tobacco products during phase 1 and then only to tobacco-flavored or nonflavored products during phase 2 will provide direct evidence for this scenario.

Last, participants will be instructed to use the research products ad libitum in their natural environment and are allowed to use other tobacco products as they wish. This is intended to enhance external validity relative to studies that may restrict use of research or other tobacco product use or be conducted in a highly controlled laboratory environment.

### Limitations

One potential study limitation is that blinding is not feasible. Consequently, participants are aware of their group assignment. We use expired-air CO as our primary outcome to minimize potential biases in participants’ self-reported cigarette consumption. Another possible limitation is the relatively short study duration (ie, a total of 6 weeks). This study design aspect was a result of balancing feasibility and available resources while also providing sufficient time for participants to achieve a meaningful reduction in their cigarette consumption. Due to the relatively short study duration, we chose at least a 50% reduction in CO values as one of our primary outcomes—and abstinence as one of our secondary outcomes—considering we may not be able to observe the full course of smoking cessation. Previous studies using the same research products have shown measurable changes in smoking behavior in a similar timeframe [13]. Finally, this study uses a nonprobability sample, a common challenge of clinical trials. We try to mitigate these limitations by setting population quotas to align the sample with the adults who smoke cigarettes population in key demographic characteristics.

## Abbreviations

CO: carbon monoxide
CFR: Code of Federal Regulations
FDA: Food and Drug Administration
GCP: Good Clinical Practice
HPHC: harmful and potentially harmful constituent
ICF: informed consent form
IRB: institutional review board
NP: nicotine pouch
SMART: sequential, multiple assignment, randomized trial
